# Editorial: Precision medicine in veterinary oncology: volume II

**DOI:** 10.3389/fvets.2023.1219963

**Published:** 2023-07-21

**Authors:** Carlos Eduardo Fonseca-Alves, Renée Laufer-Amorim, Maria Lucia Zaidan Dagli, Chiara Palmieri

**Affiliations:** ^1^Institute of Health Sciences, Paulista University, Bauru, Brazil; ^2^Department of Veterinary Surgery and Animal Reproduction, São Paulo State University, Botucatu, Brazil; ^3^Department of Veterinary Clinic, São Paulo State University, Botucatu, Brazil; ^4^Department of Pathology, School of Veterinary Medicine and Animal Science, University of São Paulo, São Paulo, Brazil; ^5^School of Veterinary Science, The University of Queensland Gatton Campus, Gatton, QLD, Australia

**Keywords:** canine, cancer, precision oncology, feline, predictive markers

Cancer is one of the most common causes of death in dogs, with an incidence 10 times higher in this species compared to humans (1). In the United States, it is estimated that 4 million of dogs and cats develop cancer each year (2). Although cancer data are not always available, this scenario is not different in other countries. To improve patients' care and develop new diagnostic and therapeutic tools, it is necessary to broaden our knowledge on the molecular aspects of the different cancer subtypes in companion animals.

Most recently, we have experienced unprecedented advances in more accessible molecular biology technologies even in veterinary oncology and, consequently, a dramatic improvement in diagnostic tests for cancer. Numerous publications have focused on molecular diagnosis and targeted therapies in the context of precision medicine (3). The use of large-scale molecular studies has provided an opportunity to understand the molecular basis of the different tumors in domestic animals and to apply this knowledge in the development of antitumor vaccines (4), specific anti-cancer monoclonal antibodies (5) and tyrosine kinase inhibitors (6) for treating different cancers in dogs and cats. Despite these advances, it is still challenging to determine which patient will benefit from specific treatments and, therefore, surgery and antineoplastic chemotherapy are still the main treatment modalities for oncological patients in veterinary medicine (3).

Another important feature of veterinary oncology is the comparative aspects with many tumors in domestic animals considered unique models to study neoplasms in humans. Animals develop spontaneous tumors with the same histological subtypes and similar molecular changes as in humans (7). Comparative Oncology has become such a paradigm in human cancer research that the US National Institute of Cancer, linked to the National Institute of Health (NIH), has created a specific program on Comparative Oncology, entirely dedicated to the comparative study of cancer in companion animals and humans. To give a comprehensive overview of the new advances in veterinary oncology and how they are assisting critical research for cancer cure both in animals and humans, this Research Topic has compiled original research and review articles that provide the most recent progresses in precision oncology applied to veterinary cancer research.

Tellado et al. provided very interesting guidelines for the standardization of the applications of electrochemotherapy (ECT) for treating superficial tumors in veterinary patients. This novel research article includes a detailed description on the use of ECT in cancer patients and practical tips to improve accuracy and outcomes for beginner users.

Martinucci et al. investigated the microRNA profile of human prostate cancer (PC) cell lines with a specific focus on fibronectin modulation. Fibronectin is an extracellular matrix glycoprotein, and its dysregulation is associated with modulation of several coding genes. Fibronectin dysregulation is likely to be important in PC development and progression and this was demonstrated by Martinucci et al. after exposure of human PC cell to fibronectin. Among the pathways dysregulated by this glycoprotein, the alteration of the PI3K/AKT/mTOR pathway seems to be predominant.

Mammary Gland Tumor (MGT) and Urothelial Carcinoma (UC) are the two tumors with high comparative significance examined by Vieira et al. and Govoni et al., respectively, and included in this Research Topic. In the former, the authors demonstrated a strong association between COX-2 protein and gene expression and different prognostic factors in canine and human mammary invasive micropapillary carcinoma. On the other hand, Govoni et al. investigated Caveolin-1, GATA-3, and Ki67 protein expression in canine urothelial carcinoma, highlighting a positive correlation of GATA-3 and Caveolin-1 expression with mitotic count, as well as an association between histopathological subtype and tumor grade.

Fu et al. performed a retrospective study comparing side effects and outcomes of three-dimensional conformal vs. intensity-modulated radiation therapy for the palliative-intent treatment (4 Gy × 5 daily fractions) of canine intranasal tumors. Radiation therapy is considered the first-line therapy for nasal tumors-affected dogs, therefore, studies comparing different radiation therapy protocols are pivotal for identifying new therapeutic strategies.

Finally, Marchi et al. reviewed all the information about the relationship between obesity, inflammation and development of cancer in dogs. Although the association of cancer with obesity is still poorly explored in animals, this review provided intriguing evidence of the pathophysiological mechanisms underlying obesity and carcinogenesis and the dangerous perspective of cancer occurrence in overweight canine patients.

Altogether, these manuscripts bring new knowledge to the field of precision medicine oncology in canine patients that may pave the way for a broader use of this strategy in veterinary oncology practice. The investigation of precision medicine approaches in veterinary oncology thus continues to be essential for a better approach of animals with cancer.

## Author contributions

All authors listed have made substantial contributions to the Research Topic, approved it for publication, contributed equally to this editorial, and approved the submitted version.
